# From discrimination to growth? Student stress in the association between perceived discrimination and posttraumatic growth among African international students in South Africa

**DOI:** 10.1371/journal.pone.0348269

**Published:** 2026-04-28

**Authors:** Juliette Bih Nkarenbi, Erhabor S. Idemudia, Lawrence Ejike Ugwu

**Affiliations:** Faculty of Humanities, North-West University, Mafikeng, South Africa; Tianjin University, CHINA

## Abstract

**Background:**

International students relocating across borders often face perceived discrimination, a form of cumulative adversity that may undermine well-being and may also be relevant to growth-related outcomes. Little is known about how perceived discrimination is associated with posttraumatic growth (PTG), and whether student stress helps explain that association, among African international students in South Africa.

**Methods:**

We surveyed 781 non-South African students aged 18–49 (54% men, 46% women) enrolled at four public universities across Gauteng, North West, Western Cape, and Free State. Perceived discrimination was assessed with the relevant subscale of the Acculturative Stress Scale for International Students; student stress was measured with four subscales (academic, interpersonal, physical and environmental) of the Student Stress Inventory; and PTG was measured with five subscales (personal strength, new possibilities, improved relationships, spiritual growth and appreciation of life) of the Posttraumatic Growth Inventory. Parallel multiple-mediator models and quadratic regression were used to estimate indirect associations and nonlinear patterns, adjusting for age and gender.

**Results:**

Perceived discrimination was moderately and positively associated with all student stress domains. It was not directly associated with most PTG facets, except for a small positive association with personal strength. Total indirect associations through student stress were nonsignificant; however, specific indirect paths diverged. Interpersonal stress showed positive indirect associations with improved relationships, new possibilities, and personal strength, whereas academic and environmental stress showed negative indirect associations with several PTG facets. Small positive quadratic terms indicated U-shaped associations between perceived discrimination and improved relationships, new possibilities, and personal strength, with the lowest estimated PTG values occurring at modestly low levels of discrimination.

**Conclusions:**

Perceived discrimination was associated with PTG through distinct student-stress patterns. Academic and environmental stress showed negative associations with several PTG facets, whereas interpersonal stress showed positive associations with some PTG facets. These findings should not be interpreted as suggesting that discrimination is beneficial; rather, they indicate heterogeneous responses to adversity. Interventions should prioritise reducing discrimination and related stressors while strengthening supportive resources for international students in South Africa.

## Introduction

South Africa has emerged as a regional hub for higher education, attracting students from across sub-Saharan Africa [1–3]. Transitioning into a new sociocultural and academic environment often entails acculturative stress: psychological, social, and somatic strain that accompanies intercultural contact (e.g., language barriers, academic demands, discrimination, loss of support) [4,5]. Contemporary accounts treat acculturation as a dual cultural–psychological process, defining acculturative stress as the discomfort produced by the pressures of adaptation (Berry’s framework), a view echoed in recent African scholarship on international students in Ghana [6]. Acculturative stress reliably forecasts elevated psychological distress among international students, including depressive symptoms [7], while social support serves as a robust protective factor [8]. Longitudinal evidence also indicates that acculturative stress and adjustment evolve during the first year abroad, underscoring the need to model dynamic student experiences [9].

However, crossing borders exposes international students to perceived discrimination, which is the subjective experience of being treated unfairly due to nationality, race, language, or cultural background [10]. Earlier qualitative and quantitative research indicates that discrimination arises from language barriers, lifestyle differences, and cultural conflicts, thereby depriving students of meaningful contact with locals and reinforcing feelings of exclusion and loneliness [10]. Such experiences are distinct from general acculturative stress but remain integral to adaptation. Recent African scholarship echoes this view, noting that acculturative stress among Ghanaian students encompasses psychological, social, and somatic strains that diminish mental health and well-being [6].

Evidence consistently links perceived discrimination to adverse mental health outcomes. International students who report discrimination display higher levels of depression, anxiety and sadness [11], and discrimination predicts homesickness, loneliness and reduced psychological adjustment among foreign students in China [10,12]. A multi-site survey of U.S. international students during the COVID-19 pandemic found that higher discrimination predicted lower positive emotions and reduced perceived social support, and that positive emotions were directly tied to both psychological distress and physical health [13]. Similarly, a recent German study of university students reported that discrimination fosters feelings of insecurity, stress and anxiety and is associated with poorer academic performance [14]. These converging findings underline perceived discrimination as a potent risk factor for the well-being of international students.

Despite its detrimental effects, adversity may be associated with posttraumatic growth (PTG), positive psychological change in personal strength, relationships, spirituality and appreciation of life following highly challenging life circumstances. PTG was originally formulated in relation to discrete traumatic events, but later scholarship has also considered whether growth-related changes may arise in the context of cumulative or chronic adversity. In student and migration contexts, such adversity may involve repeated stressors that accumulate over time rather than a single event. Among international students, a meaning-making model revealed that acculturative stressors serve as risk factors, whereas sense-making coping and re-examination of core beliefs partially mediate post-migration growth [15]. In a large multi-country university sample, resilience fully mediated the link between negative stress (distress) and PTG and partially mediated the link between positive stress and PTG [16], highlighting the importance of intervening mechanisms rather than simple direct stress–growth associations.

The functional form of the discrimination–growth relationship remains a topic of debate. Stress research suggests that curvilinear patterns may occur: very low stress provides little impetus for cognitive engagement, whereas extreme stress overwhelms coping resources; growth-related outcomes may be more likely at intermediate levels [17,18]. If perceived discrimination functions similarly, linear models may conceal meaningful nonlinear associations. At the same time, interpreting such patterns requires caution: any association between discrimination and PTG would reflect how some students respond to adversity, not a beneficial property of discrimination itself. Furthermore, the mechanisms through which discrimination may relate to growth are poorly understood. Student stress (comprising academic, interpersonal, physical and environmental demands) may be an important explanatory pathway. Discrimination may exacerbate academic challenges (e.g., language difficulties, perceived unfairness), strain interpersonal relationships, increase physical complaints, and amplify environmental hassles such as financial strain. Student stress, in turn, has been linked to lower PTG in previous research, suggesting that it may help account for associations between discrimination and growth. However, empirical work explicitly testing these indirect associations is scarce, particularly in African contexts.

Despite a growing global literature, African evidence remains limited and often focuses on general student mental health rather than mechanisms linking acculturative stress to growth. Recent South African evidence shows a substantial mental-health burden among university students: a national survey across 17 public universities reported 30-day prevalence estimates of 16.3% for mood disorders and 37.1% for anxiety disorders [19]. In contrast, provincial/discipline-specific studies report high levels of probable depression and anxiety among undergraduates [20]. In parallel, policy analyses and institutional guidance advocate for inclusive, better-resourced services, including provisions responsive to international students [21,22]. Recent scholarship also outlines the delivery components for inclusive mental health support for international students [23]. Regional qualitative studies document rich, context-specific stressors (financial pressures, environmental hassles, stereotyping) and the buffering role of social networks, reinforcing the salience of student-stress contexts for African sojourners [6].

### Study rationale and objectives

The present study addresses these gaps by focusing on African international students in South Africa, conceptualising perceived discrimination as the focal independent variable. We model four student-stress domains (academic, interpersonal, physical, and environmental) as explanatory variables in the association between perceived discrimination and the five PTG facets (personal strength, new possibilities, improved relationships, spiritual growth, and appreciation of life). Building on prior mediation findings [15,16] and curvilinearity evidence [17,18], we test whether (a) perceived discrimination is associated with higher student stress; (b) student stress is associated with PTG; (c) specific indirect associations link discrimination to PTG via student stress; and (d) the discrimination–PTG relation is linear or quadratic. By integrating indirect-association and nonlinear analyses in an under-researched African setting, our study examines whether perceived discrimination, conceptualised here as a form of cumulative adversity rather than a discrete traumatic event, is statistically associated with PTG and through which student-stress domains these associations are observed.

## Method

### Design

We conducted a cross-sectional, quantitative survey of international (non-South African) students enrolled at four public universities in South Africa. Primary aims were to (a) describe zero-order associations among perceived discrimination, student-stress domains, and posttraumatic growth (PTG); (b) estimate parallel multiple-mediator models in which student-stress domains account for the association between perceived discrimination and PTG; and (c) examine quadratic (curvilinear) associations of perceived discrimination with PTG facets. Analyses were planned a priori; perceived discrimination was the focal independent variable, student-stress domains were modelled as intervening variables, and PTG facets were the outcomes. With N = 781, the study had adequate power to detect small correlations and path coefficients.

### Setting and context

The sampling frame comprised public universities in four provinces (Gauteng, North West, Western Cape, and Free State), representing diverse urban, peri-urban, and coastal contexts in South Africa. Data were collected online via institutionally distributed survey links.

### Participants

#### Eligibility and recruitment.

The inclusion criteria were students aged 18 years and above, currently registered at one of the selected universities, and non-South African nationality (screened at the time of consent). South African nationals and incomplete/invalid responses were excluded. Recruitment used International Office mailing lists, campus posters, and official web posts. To balance institutional representation, we used site-balanced, non-proportional quotas (target ≈ 200 per site).

#### Sample characteristics.

A total of 781 students provided analysable data (age: *M* = 28.22, *SD* = 10.16, range 18–49). Gender: 422 men (54.0%) and 359 women (46.0%). Participants represented 36 African countries; the largest groups were Nigeria (12.3%), Uganda (6.5%), Zambia (5.6%), Ghana (5.5%), Kenya (5.5%), Cameroon (5.9%), DRC (5.4%), and Namibia (5.1%) (each remaining country ≤ 4%).

#### Ethics and consent.

The study received ethics approval from the North-West University Health Research Ethics Committee (NWU-HREC), Faculty of Health Sciences (ethics number NWU-00052–22-S1; 06/06//2022). Recruitment took place across four South African public universities through institutionally distributed survey links, starting on 15/07/2022 and concluding on 30/01/2023. Eligible participants were currently registered non-South African students aged 18 years or older who provided written, electronic informed consent prior to participation. Participation was voluntary, with confidentiality/anonymity safeguards in place; a site-based clinical psychologist referral pathway was provided, and participants received R25 airtime as a token of appreciation. Data are stored on password-protected servers/in locked cabinets and retained for seven years in accordance with policy.

### Measures/materials

#### Demographics.

Age (years), gender (coded 0 = male, 1 = female), and country of origin were recorded for descriptive and control purposes.

#### Perceived discrimination.

Perceived discrimination (primary predictor) was assessed using the Perceived Discrimination subscale of the Acculturative Stress Scale for International Students (ASSIS) [24]. This subscale comprises 8 items assuring students’ subjective sense of unfair or negative treatment due to their nationality, race, language, or cultural background (e.g., “I am treated differently in social situations”). Items are rated on a 5-point scale (1 = *Strongly disagree* to 5 = *Strongly agree*) and summed to yield scores ranging from 8 to 40, with higher scores indicating greater perceived discrimination.

#### Student stress.

The Student Stress Inventory (SSI; [25]) contains 40 items (1 = *Never* to 4 = *Always*) forming four 10-item subscales: Physical, Interpersonal, Academic, and Environmental stress. Higher scores indicate greater stress.

#### Posttraumatic growth (PTG).

The Posttraumatic Growth Inventory (PTGI; [26]) consists of 21 items that assess five facets of posttraumatic growth: Personal Strength, New Possibilities, Improved Relationships, Spiritual Growth, and Appreciation of Life. Items were summed/averaged according to the manual to create facet scores (higher values indicating greater PTG).

#### Procedure.

After institutional permissions, eligible students accessed an online eligibility screen and consent page; those who consented completed the questionnaire. Participation was self-paced and remote. A clinical support pathway was outlined on the consent form and during the debrief.

#### Data management and screening.

Records failing eligibility or exhibiting excessive missingness were removed before analysis (final N = 781). Remaining missingness was minimal and handled via listwise deletion at the scale level. Continuous predictors were mean-centred before constructing polynomial terms; multicollinearity diagnostics indicated acceptable levels (VIFs < 5). All scoring followed instrument guidelines.

### Statistical analysis

All analyses were conducted in SPSS v30 and SmartPLS v4. We first reported descriptive statistics and zero-order Pearson correlations among all focal variables. To examine indirect associations, we estimated parallel multiple-mediator models with perceived discrimination (X) predicting each PTG facet (Y) through the four SSI subscales (M), controlling for age and gender on both mediators and outcomes. We report standardised coefficients for (a) specific indirect associations (from perceived discrimination through each SSI domain to each PTG facet), (b) total indirect associations, (c) direct associations (perceived discrimination with each PTG facet net of the mediators), and (d) total associations, along with R² for mediators/outcomes and Cohen’s f² effect sizes. Statistical inference used bootstrap percentile 95% confidence intervals based on 5,000 resamples; effects whose CIs excluded zero were deemed significant. Given the cross-sectional design, these models are interpreted as statistical decompositions of association rather than evidence of temporal or causal pathways.

Because theory suggests that discrimination–growth relations may be nonlinear, we fitted hierarchical polynomial regressions for each PTG facet, entering centred perceived discrimination at Step 1 and (perceived discrimination)² at Step 2. We report standardised coefficients, turning points (x⋆ = −b₁/2b₂, in SD units), and simple slopes at ±1 SD (slope = b₁ + 2b₂·x). Unless otherwise noted, tests were two-tailed with α = .05, and interpretation emphasised confidence intervals, effect sizes, and caution in reading quadratic patterns.

## Results

Overall, the reliability statistics indicate that all constructs display acceptable to high internal consistency. Composite reliability values further confirm the reliability of the measurement models, while the AVE values demonstrate adequate convergent validity. These findings suggest that the instruments used are psychometrically sound [27,28]. The results provide confidence that the observed relationships in the structural model are not artefacts of measurement error but reflect substantive associations among the constructs.

Table 1 shows that age correlated negatively with most PTG domains. In contrast, men showed small associations with perceived discrimination and environmental and physical stress domains, indicating slightly lower discrimination and stress scores among women on those variables. Overall, the pattern supports modelling student stress as a correlate of perceived discrimination and a generally adverse correlate of PTG, while direct zero-order associations between perceived discrimination and PTG are minimal.

**Table 1:** Descriptive statistics and intercorrelations among study variables.

#	Variable	M	SD	α	CR	AVE	1	2	3	4	5	6	7	8	9	10	11	12
1	**Age**	28.22	10.16	—	—	—	—											
2	**Gender** (0 = Male, 1 = Female)			—	—	—	−.11**	—										
3	**Perceived discrimination (PD)**	29.17	5.30	0.93	0.94	0.66	.06	−.17**	—									
4	**Physical (SS)**	17.05	4.77	0.84	0.88	0.52	.02	−.08*	.40**	—								
5	**Interpersonal (SS)**	17.68	3.86	0.75	0.77	0.58	−.08*	−.03	.40**	.68**	—							
6	**Academic stress (SS)**	16.16	5.33	0.92	0.93	0.59	.05	−.06	.43**	.58**	.68**	—						
7	**Environmental (SS)**	15.66	5.43	0.91	0.92	0.54	−.04	−.10**	.43**	.50**	.59**	.80**	—					
8	**Personal strength (PTG)**	17.70	2.29	0.73	0.77	0.51	−.17**	.05	−.02	−.01	.04	−.11**	−.11**	—				
9	**New possibilities (PTG)**	22.24	2.77	0.88	0.92	0.74	−.19**	.05	−.03	−.01	.05	−.07*	−.07*	.92**	—			
10	**Improved relationships (PTG)**	30.80	4.00	0.89	0.92	0.75	−.21**	.04	−.03	.03	.10**	−.09*	−.09*	.91**	.91**	—		
11	**Spiritual growth (PTG)**	8.88	1.11	0.77	0.89	0.81	−.02	.06	−.01	−.01	.05	−.04	−.09*	.80**	.82**	.81**	—	
12	**Appreciation of life (PTG)**	13.48	1.58	0.79	0.88	0.70	−.13**	−.01	−.02	−.02	−.01	−.09*	−.11**	.82**	.86**	.82**	.75**	—

**Note.** *p* < .05 (*), *p* < .01 (**). α - Cronbach’s α values are reported for multi-item scales only; α values close to 1.0 indicate stronger internal consistency. CR-Composite reliability and average variance extracted (AVE) reflect reliability and convergent validity; values above.70 (ρ) and.50 (AVE) are desirable. Gender (dummy coded ‘0’-Male, ‘1’-Female); SS-student stress; PTG- Posttraumatic Growth.

Perceived discrimination (PD) was moderately and positively associated with all student-stress domains (physical, interpersonal, academic, and environmental), but was not significantly related to any PTG domains. Student-stress domains were significantly correlated with PTG, with one notable exception: interpersonal stress, which was associated with improved relationships (r = .10, p < .01).

In a parallel multiple-mediator model (see Table 2), the total indirect associations between perceived discrimination (PD) and posttraumatic growth (PTG) through student stress were not significant for any PTG domain. The only significant direct association was a small positive association between PD and personal strength (b = .101, 95% CI [.011,.192], p = .029).

**Table 2:** Mediation of perceived discrimination (X = PD) on PTG facets via student stress domains.

X	Y (PTG Domains)	Mediator (M)	Specific indirect b [95% CI]	Total indirect b [95% CI]	Direct b [95% CI]	Total effect b
PD	appreciation life	Academic stress	−0.026 [−0.087, 0.038]	−0.040 [−0.098, 0.018]	0.074 [−0.013, 0.161]	0.034
		Environmental	−0.065 [−0.123, −0.015]			
		Interpersonal	0.047 [−0.007, 0.102]			
		Physical	0.004 [−0.031, 0.040]			
PD	improved relationships	Academic stress	−0.085 [−0.159, −0.007]	−0.005 [−0.063, 0.052]	0.086 [−0.001, 0.174]	0.081
		Environmental	−0.056 [−0.121, 0.003]			
		Interpersonal	0.127 [0.070, 0.185]			
		Physical	0.009 [−0.030, 0.048]			
PD	New possibilities	Academic stress	−0.083 [−0.151, −0.011]	−0.015 [−0.075, 0.045]	0.059 [−0.026, 0.145]	0.044
		Environmental	−0.027 [−0.086, 0.027]			
		Interpersonal	0.098 [0.039, 0.159]			
		Physical	−0.004 [−0.042, 0.032]			
PD	personal strength	Academic stress	−0.077 [−0.143, −0.004]	−0.030 [−0.089, 0.024]	0.101 [0.011, 0.192]	0.069
		Environmental	−0.059 [−0.119, −0.004]			
		Interpersonal	0.099 [0.046, 0.154]			
		Physical	0.004 [−0.038, 0.045]			
PD	spiritual growth	Academic stress	0.004 [−0.046, 0.054]	−0.024 [−0.082, 0.032]	0.082 [−0.005, 0.172]	0.058
		Environmental	−0.092 [−0.144, −0.044]			
		Interpersonal	0.082 [0.030, 0.134]			
		Physical	−0.017 [−0.055, 0.019]			

Note. Bolded entries indicate 95% CIs excluding zero. Total effect = Direct + Total indirect.

Although the total indirect associations were not significant, several specific indirect paths were statistically significant and in opposite directions. Interpersonal stress showed positive indirect associations from PD to PTG (improved relationships: b = .127, 95% CI [.070,.185], p < .001; new possibilities: b = .098, 95% CI [.039,.159], p = .001; personal strength: b = .099, 95% CI [.046,.154], p < .001; spiritual growth: b = .082, 95% CI [.030,.134], p = .002). In contrast, academic and environmental stress showed negative indirect associations (academic → improved relationships: b = −.085, 95% CI [−.159, − .007], p = .028; academic → new possibilities: b = −.083, 95% CI [−.151, − .011], p = .021; academic → personal strength: b = −.077, 95% CI [−.143, − .004], p = .031; environmental → appreciation of life: b = −.065, 95% CI [−.123, − .015], p = .018; environmental → personal strength: b = −.059, 95% CI [−.119, − .004], p = .044; environmental → spiritual growth: b = −.092, 95% CI [−.144, − .044], p < .001). Physical stress did not yield significant indirect effects. The coexistence of positive (interpersonal) and negative (academic/environmental) specific paths likely offsets at the aggregate level, accounting for the nonsignificant total indirect effects.

Quadratic terms clarified the form of the PD–PTG relations (see Table 3). The positive quadratic effect was significant for improved relationships (b₂ = .118, 95% CI [.068,.172], p < .001), new possibilities (b₂ = .083, 95% CI [.024,.144], p = .007), and personal strength (b₂ = .099, 95% CI [.039,.160], p = .002), but not for appreciation of life (b₂ = .059, 95% CI [−.011,.130], p = .099) or spiritual growth (b₂ = .069, 95% CI [−.008,.148], p = .090). Positive quadratic coefficients imply upward curvature (U-shape) when considered alongside the linear term. Given the modest size of these effects, they are interpreted cautiously.

**Table 3:** Quadratic effects (QE) of perceived discrimination (PD) on PTG facets, with turning points and simple slopes.

Y (PTG domains)	Linear PD → Y b_1_ [95% CI]	Quadratic PD² → Y b_2_ [95% CI]	Turning point x* (SD)	Slope at −1 SD	Slope at +1 SD
appreciation life	0.074 [−0.013, 0.161]	0.059 [−0.011, 0.130]	−0.627	−0.044	0.192
improved relationships	0.086 [−0.001, 0.174]	0.118 [0.068, 0.172]	−0.364	−0.150	0.322
New possibilities	0.059 [−0.026, 0.145]	0.083 [0.024, 0.144]	−0.355	−0.107	0.225
personal strength	0.101 [0.011, 0.192]	0.099 [0.039, 0.160]	−0.510	−0.097	0.299
spiritual growth	0.082 [−0.005, 0.172]	0.069 [−0.008, 0.148]	−0.594	−0.056	0.220

Note. Bold = 95% CI excludes zero. Standardized coefficients; bootstrap 95% CIs; two-tailed p. Turning point x*=−b1/(2b_2_) is in SD units of PD; simple slopes are dY/dx = b_1_ + 2b_2_x at x=±1 SD.

Turning points (in SD units of PD) fell slightly below the mean—about −0.36 SD for improved relationships, −0.36 SD for new possibilities, and −0.51 SD for personal strength—indicating that the estimated functions reached their minima at modestly low PD and then rose across higher observed levels of PD. Simple-slope diagnostics showed that the instantaneous slope was negative at low PD (−1 SD) and positive at high PD (+1 SD) for these significant outcomes (e.g., improved relationships: slope−1SD = −.150, slope+1SD = .322; personal strength: − .097 vs..299; new possibilities: − .107 vs..225).

Interpretation. Beyond the indirect-association models, the PD–PTG association appears nonlinear for several facets: at higher observed levels of PD, estimated PTG values were higher for improved relationships, new possibilities, and personal strength than at lower levels. This pattern is consistent with, but does not directly demonstrate, challenge-related growth accounts. It should not be interpreted as evidence that discrimination is beneficial; rather, it suggests that the discrimination–PTG association may be non-linear and heterogeneous across students. No reliable nonlinearity was observed for appreciation of life or spiritual growth.

Endogenous mediators were moderately explained (R^2^ = .158–.188; adjusted R^2^ = .157–.187), whereas PTG facets showed small, explained variance (R^2^ = .025–.076; adjusted R^2^ = .017–.069). Effect-size indices (f^2^; Cohen) indicated trivial direct contributions of PD to PTG (all f^2^ ≤ .006). Among mediators, interpersonal stress showed small effects (improved relationships f^2^ = .045; new possibilities f^2^ = .026; personal strength f^2^ = .027; spiritual growth f^2^ = .018), with smaller contributions from environmental (spiritual growth f^2^ = .016; appreciation of life f^2^ = .008) and academic stress (improved relationships f^2^ = .012; new possibilities f^2^ = .011; personal strength f^2^ = .010). The quadratic term for PD contributed small additional variance for several facets (e.g., improved relationships f^2^ = .027; new possibilities f^2^ = .013; personal strength f^2^ = .019).

## Discussion

### Summary of main findings

This study examined how perceived discrimination (a key domain of acculturative stress) is associated with student stress and posttraumatic growth (PTG) among African international students in South Africa. By modelling four student-stress domains (physical, interpersonal, academic and environmental) as parallel mediators between perceived discrimination and five PTG facets, we tested both linear and quadratic associations in a large cross-sectional sample. The results reveal four main patterns. First, perceived discrimination was moderately and positively associated with all student stress domains but showed negligible direct relations with most PTG facets. Second, the total indirect associations between discrimination and PTG via student stress were nonsignificant; however, specific indirect paths emerged in opposite directions: interpersonal stress showed positive indirect associations with improved relationships, new possibilities, personal strength and spiritual growth, whereas academic and environmental stress showed negative indirect associations with several PTG facets (e.g., lower appreciation of life and personal strength). Third, small quadratic (U-shaped) associations were observed for improved relationships, new possibilities, and personal strength, with the lowest estimated values occurring at modestly low levels of discrimination and higher estimated values at higher observed levels. Fourth, the overall pattern suggests heterogeneity in how discrimination, student stress and PTG are related, rather than a single uniform pathway.

### Interpretation in light of prior research

The pattern whereby perceived discrimination correlates strongly with student stress, yet only weakly with PTG, is consistent with prior work documenting the psychological costs of discrimination. International students who report discrimination experience elevated depression, anxiety and sadness [12], as well as higher loneliness and poorer psychological adjustment [10]. In a multi-site U.S. survey, discrimination was linked to lower positive emotions and weaker perceived social support, which in turn predicted worse psychological and physical health [13]. Our findings align with this literature: discrimination appears to be linked to PTG primarily through stress-related correlates rather than strong direct associations. That said, the absence of a direct discrimination–PTG association does not imply that discrimination is irrelevant for growth. Instead, growth-related outcomes may depend on how discriminatory experiences are processed and appraised, a conclusion supported by meaning-making models, in which acculturative stressors are risk factors, but sense-making coping and core belief re-examination partially mediate the link to growth [15]. We also recognise that PTG theories often emphasise subjective meaning-making and cognitive restructuring, whereas our cross-sectional quantitative models capture only statistical association patterns. Accordingly, the present analyses should be read as consistent with those processes, not as direct evidence of them.

The positive specific indirect associations involving interpersonal stress may be understood in light of rejection-identification and challenge-hindrance perspectives, but only cautiously. According to the rejection-identification model, discrimination can be associated with stronger in-group identification and support seeking within one’s community [29]. Qualitative research on international students also suggests that some interpersonal difficulties are retrospectively reframed as learning experiences [10]. In that sense, interpersonal stress may sometimes coincide with efforts to invest in relationships and derive meaning from adversity. However, our data do not show that discrimination or interpersonal strain is inherently growth-promoting; rather, they suggest that relational stress may coexist with adaptive processing for some students. By contrast, academic and environmental stress appear more consistently aligned with hindrance-type burdens that consume cognitive and emotional resources and may undermine adaptation [10].

The observed quadratic effects further illuminate this dynamic. For improved relationships, new possibilities and personal strength, the functions were U-shaped, with minima slightly below the mean discrimination score and higher estimated values at higher observed discrimination levels. One interpretation is that low levels of discrimination may be insufficient to trigger deeper reflection, whereas more intense cumulative adversity may coincide with greater meaning-making or support mobilisation for some students. Yet this interpretation must remain tentative. The quadratic effects were modest, the design was cross-sectional, and the findings should not be read as implying an optimal or desirable level of discrimination. Rather, the results suggest that the association between discrimination and some PTG domains may be non-linear and contingent on appraisal, coping resources, and context.

### Theoretical and practical implications

These findings contribute to acculturation and stress-growth scholarship in three ways. First, they show that student-stress domains are not interchangeable: interpersonal stress was linked to positive indirect associations with some PTG domains, whereas academic and environmental stress showed negative indirect associations. Second, the study extends discussion of PTG beyond discrete trauma by examining perceived discrimination as a form of cumulative adversity; in this framing, PTG is considered a possible response to chronic strain rather than proof that the strain is constructive. Third, the findings underline the need to integrate social-cognitive and contextual variables (for example, meaning-making, coping, social support, and identity processes) into future models to explain why some students report growth-related outcomes alongside adversity. Because the indirect and quadratic effects were modest and the total indirect effects were nonsignificant, these contributions should be interpreted cautiously.

Practically, universities and policymakers should prioritise reducing discriminatory experiences and strengthening supportive social networks. The findings do not imply that discrimination or interpersonal strain should be tolerated as developmental tools. Rather, institutions should minimise academic and environmental burdens (for example, unclear academic expectations, language barriers, bureaucratic hurdles and housing or financial difficulties) while ensuring access to culturally sensitive counselling, peer-support services, mentoring, and inclusive campus practices. Such supports may help students process discriminatory encounters, draw on social resources, and engage in meaning-focused coping without normalising harm.

### Limitations and strengths

This study is subject to several limitations. The cross-sectional design precludes causal inference; the mediation analyses should therefore be interpreted as statistical decompositions of association rather than evidence of temporal or causal pathways. Reverse or reciprocal relationships are also plausible: students with greater existing PTG or personal resources may appraise discrimination differently, and stress and PTG may influence one another over time. The use of self-report measures may introduce common-method bias and social desirability effects; combining surveys with qualitative interviews or behavioural data could enrich future analyses. Although the Perceived Discrimination subscale of the ASSIS captures unfair treatment across multiple domains, it does not differentiate between chronic microaggressions and acute discriminatory events; refining the measurement of discrimination could yield more nuanced insights. The sample, although large and geographically diverse, was restricted to African international students at four South African universities, limiting generalizability to other regions or cultural groups. We controlled for age and gender but not for other potentially relevant variables, including socioeconomic status, prior adversity, coping style, resilience, social support, identity processes, length of stay, or personality traits. Finally, the explained variance for PTG domains was modest, indicating that additional mechanisms likely contribute to growth.

Strengths of the study include its large, multi-institutional sample, which enhances statistical power and diversity; the integration of multiple stress domains and PTG facets, allowing us to parse specific pathways; and the use of PLS-SEM with bootstrapping and quadratic modelling, which provides a nuanced view of both linear and non-linear associations. The reliability and validity checks indicate that our measurement models are robust, further supporting the credibility of the findings.

### Future directions

Future research should take a longitudinal or mixed-method approach to examine how discrimination, stress, appraisal and growth-related outcomes evolve across the academic trajectory. Incorporating measures of coping strategies, social identity processes, resilience, social support, prior adversity, and meaning-making would help clarify the mechanisms through which discrimination relates to growth or stagnation. Alternative model structures, including reciprocal or reverse pathways, should also be evaluated. Comparative studies across different host countries, disciplines and student subgroups could illuminate contextual factors that moderate these relationships. Intervention studies could assess whether programmes aimed at reducing discrimination and strengthening support systems improve student well-being and growth-related outcomes. Qualitative studies could further explore students’ narratives of discrimination and growth to capture the lived experience of these processes.

## Conclusion

In summary, perceived discrimination was associated with student stress and with PTG-related outcomes in heterogeneous ways among African international students. Academic and environmental stress showed negative indirect associations with several PTG domains, whereas interpersonal stress showed positive indirect associations with some PTG domains, even though total indirect effects were nonsignificant. Small U-shaped associations were also observed for improved relationships, new possibilities and personal strength. These findings should not be interpreted as suggesting that discrimination is beneficial. Rather, they indicate that responses to cumulative adversity may vary across stress domains and students, underscoring the need for institutions to reduce discrimination and strengthen supportive conditions for international students.

## Supporting information

S1 Data De-identified dataset underlying the analyses reported in this manuscript. (CSV)
